# Calcium-Reduced Micellar Casein Concentrate—Physicochemical Properties of Powders and Functional Properties of the Dispersions

**DOI:** 10.3390/foods11101377

**Published:** 2022-05-10

**Authors:** Anil Kommineni, Venkateswarlu Sunkesula, Chenchaiah Marella, Lloyd E. Metzger

**Affiliations:** 1 Midwest Dairy Foods Research Center, Dairy and Food Science Department, South Dakota State University, Brookings, SD 57007, USA; akommineni@jacks.sdstate.edu (A.K.); cmarella@idahomilk.us (C.M.); lloyd.metzger@sdstate.edu (L.E.M.); 2 Idaho Milk Products, 2249 South Tiger Drive, Jerome, ID 83338, USA

**Keywords:** reduced calcium micellar casein concentrate, CO_2_ injection, ultrafiltration, functional properties, viscosity, solubility, heat stability, emulsification, foaming

## Abstract

This study aimed to examine the physicochemical properties of 30% calcium (Ca)-reduced micellar casein 80% protein powders (RC-MCC) and the functional properties of the resultant dispersions. The calcium reduction in the micellar casein (MCC) powder was achieved by subjecting the liquid micellular casein obtained from the microfiltration of pasteurized skim milk to carbon dioxide (CO_2_) treatment before and during ultrafiltration. The CO_2_ injection was controlled to obtain a 0 and 30% reduction in calcium in the C-MCC (control) and RC-MCC powders, respectively. The MCC powders were tested for physicochemical properties such as chemical composition, particle size distribution, and bulk density. The MCC powders were reconstituted in deionized water to test the functional properties of the dispersions, i.e., solubility, viscosity, heat stability, emulsifying capacity, emulsion stability, foam capacity, and foam stability. The CO_2_ injection did not result in any significant differences in the composition except mineral contents, particularly calcium. The particle size and bulk density of RC-MCC powders were significantly (*p* < 0.05) lower than control powders. The RC-MCC powder dispersions showed increased heat stability compared to control, whereas no significant changes in viscosity and emulsification capacity were observed between the two dispersions. However, the emulsion stability and foam stability of RC-MCC dispersions were significantly lower than C-MCC dispersions. This study showed that by utilizing a novel microfiltration–CO_2_ injection–ultrafiltration process, 30% calcium-reduced MCC powder was commercially feasible. This research also provides a detailed understanding of the effect of calcium reduction on the functional properties of resultant MCC dispersions. It showed that calcium reduction could improve the solubility of the powders and heat stability and foam capacity of the dispersions.

## 1. Introduction

Micellar casein concentrate (MCC) has recently received much attention because of its unique functional properties and applications in various foods [1]. Micellar casein concentrate is manufactured by microfiltration (MF) of skim milk [2,3], which partitions serum proteins, non-protein nitrogen, lactose, and serum minerals into the permeate. The resulting casein-enriched (higher casein to total protein ratio and casein to true protein) retentate is referred to as micellar casein concentrate. MCC powders (80% (wt./wt.)) are produced from this liquid micellar casein concentrate by spray drying. The casein’s general physical and compositional properties in MCC are similar to that of the native casein micelles in milk [4]. Micellar casein concentrate may be used to produce cheese, processed cheese (as a rennet casein substitute), nutritional meal replacements, whipped toppings, RTD protein drinks, and coffee whiteners, among other applications [5,6]. Additionally, they are an excellent raw material for producing bifunctional peptides [7,8,9] and for a variety of non-food applications such as coating agents [10] and glues [11]. As a result, interest in casein products and fractions has increased steadily [12].

Because MCC is a relatively new product, few detailed studies on its functionality are available. However, since MCC contains a higher proportion of casein and a lower proportion of whey proteins (WP), specific properties such as improved heat stability are assumed. Nonetheless, the general properties of casein and casein micelles have been thoroughly described [13,14]. High insolubility is one of the significant issues when using MCC. One of the most important functional properties of any dried protein powder is solubility. Protein powders tend to lose solubility because of protein–protein interactions during manufacturing and storage. The proteins need to be rehydrated to ensure optimal functionality [15]. The solubility during the rehydration of MCC powder has also been a limiting factor in its use in foods and beverages [16,17]. The strong influence of mineral content on the solubility of protein powders was studied in previous research studies [18,19,20]. In particular, the calcium content plays an important role in the solubility of micellar casein. Removal of calcium to enhance the solubility of MPCs was performed using acidification of milk and the addition of calcium chelators, and the replacement of calcium with monovalent salts has gained researchers’ interest [21]. Therefore, changing the calcium equilibrium towards a higher proportion of serum calcium has become a promising approach to minimizing protein aggregation and enhancing solubility [22,23]. The lower solubility of MCC powders that deteriorates over time has been reported and is ascribed to higher-order structural changes, such as cross-linking between casein micelles, which may involve the formation of intermolecular sheets [24]. Biochemical and physical modifications to the concentrates and powders increased the solubility index to 92.6 percent [25]. Therefore, there exists a significant opportunity to improve the functional properties of MCC, such as solubility and heat stability [26]. 

Several recent studies have detailed the chemical composition of MCC powders processed with MF [12,27,28]. MCC generally contains more than 90% casein (CN) on total protein, whey protein (WP), and soluble constituents such as lactose and soluble minerals. Minerals, lactose, and non-protein nitrogen (NPN) all significantly affect the heat and alcohol stability of MCC [1]. Due to its high CN content, MCC is expected to have excellent heat and alcohol stability. Previously, it was reported that CN is a heat-stable compound [29]. Recent studies, however, indicate that heat stability may not be as high as previously believed [30,31]. Certain pH and temperature conditions, on the other hand, can cause the CN micelles to lose their integrity, resulting in flocculation, gelation, or protein separation [32,33], and can affect the functionality of the CN micelle upon heating [30,31]. The pH, calcium content, protein concentration, urea (NPN), lactose, and SP concentration significantly affect solubility and heat stability. Changes in solubility and heat stability occur due to the constituents and ratios of milk components changing during MF or ultrafiltration (UF) operations, with diafiltration (DF) further altering the composition [34]. Sauer and Moraru, 2012, concluded that MCC is unstable at sterilization temperature. This instability increases with treatment temperature due to changes in the mineral equilibrium and partial disintegration of the CN micelle, which results in aggregation and even coagulation. Minor differences in the composition and processing of the MCC can result in significant differences in their sterilization stability. Some studies reported that drying and reconstitution of MCC decreased its resistance to UHT treatment compared to the undried concentrates [31,35]. Most of the calcium in milk is contained in casein micelles. Micellar calcium is primarily present as colloidal calcium phosphate (CCP) nanoclusters. The amount of micellar calcium can be altered by altering environmental conditions such as temperature, pH, or the addition of chelators [36,37]. The concentration of salts, particularly calcium, significantly affects the sensory and functional properties of fermented dairy products [38,39] and dairy powders [20,40]. For instance, it was reported that cheddar cheese (and processed cheeses made thereof) prepared from ultrafiltration (UF) milk retentate had a decreased melting ability due to its high calcium content [39]. Numerous researchers have found a close link between the solubility of MPC80 and its Ca content [19,41,42] leading to the hypothesis that Ca present in MPC may promote protein–protein interactions during processing and storage. Several studies have been undertaken in recent years to determine the effect of demineralization on the rehydration behavior of casein powders [43,44] or calcium content [24,45,46,47]. Mao et al., 2012 and Sikand et al., 2013 developed a novel method for mineral-modified MPC80 production by utilizing DF with varying concentrations of monovalent salts added to the DF water. Mineral-modified MPC80 produced via this process exhibited enhanced functionality and solubility than conventionally produced MPC80. In addition, [48,49] used a cation-exchange method to replace divalent ions, particularly calcium, and reported improved functional properties for the calcium-depleted MPC. Based on these previous studies demonstrating enhanced functionality of MPC powders with reduced calcium content, we hypothesized that micellar casein concentrates with reduced calcium content would exhibit similar functional benefits. 

Injecting CO_2_ into cheese milk before rennet coagulation was reported to decrease the pH of the milk and solubilize micellar calcium phosphate, altering the mineral profile of cheese made from concentrated milk [50,51]. Similarly, CO_2_ injection could lower the milk pH and solubilize micellar calcium and phosphate before and during UF, resulting in decreased calcium and mineral content of MPC [47,52]. In addition, when CO_2_ is used as an acidulant, residual CO_2_ can be easily removed via heating or vacuum, whereas other acidulants such as organic acids cannot. However, no studies to date have been reported on the use of CO_2_ to reduce calcium in MCC powders. Therefore, it was hypothesized that reduction of calcium content in MCC could improve the functional properties such as solubility and heat stability. The current study produced 30% calcium-reduced MCC powders by modifying the Marella et al., 2015 process used to manufacture 30% calcium-reduced MPCs. Marella et al., 2015 reported improved functional properties in 30% reduced-calcium MPC powders which was attributed to the formation of more soluble caseins when colloidal calcium was removed. However, the standard MCC manufacturing process uses MF only, and the dissociated casein fractions may permeate if the skim milk is acidified. An additional ultrafiltration step of liquid MCC acidification could reduce the loss of the soluble caseins. As a result, the study’s goal was to determine the effect of a 30% calcium reduction in MCC produced through a novel multistage MF-UF filtration process that utilizes carbon dioxide to acidify milk before and during the UF stage.

## 2. Materials and Methods

### 2.1. Manufacturing of MCC80 Powders

**Figure 1:**
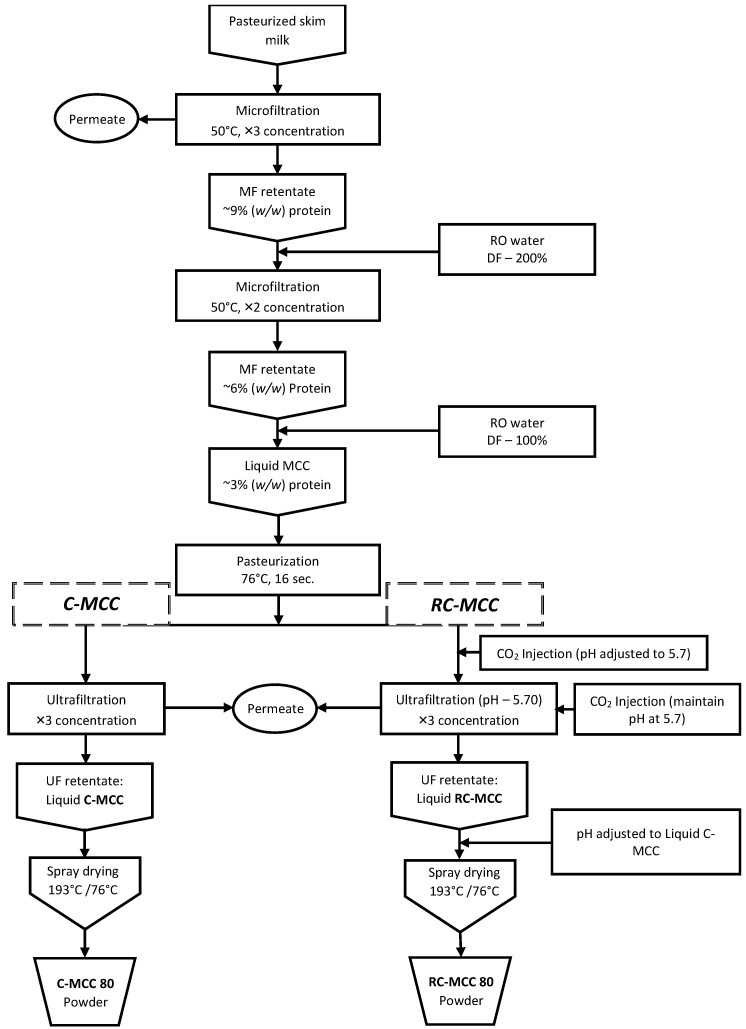
Schematic of the production of MCC 80 powders with and without calcium reduction.

Micellar casein concentrate (MCC80) powders with two levels of calcium, 0% reduction (control, C-MCC) and 30% reduction (treatment, RC-MCC), were manufactured in triplicates using a novel MF-UF method. A schematic C-MCC and RC-MCC production process is shown in Figure 1. The pasteurized skim milk was microfiltered to 3× concentration at ~50 °C and transmembrane pressure (TMP) of 0.1 MPa using a GP MF (TIA) cross-flow membrane filtration unit with ceramic membranes (GP Membralox^®^ modules, MYCRODYN_NADIR) of 0.1 µm mean pore size, 1.68 m^2^ total surface area, and 1.02 m length. The retentate from this MF step, with 9% (wt./wt.) protein, was diluted back to 3% (wt./wt.) protein using deionized water, making it a 200% DF rate. The liquid MCC thus obtained was again microfiltered (DF-MF) to 2X concentration to permeate more serum phase components and to increase the casein fraction in the retentate. The liquid MCC obtained from this DF-MF step, having 6% (wt./wt.) protein, was diluted back to 3% (wt./wt.) protein, pasteurized (76 °C/16 s), and stored at 4 °C for further ultrafiltration processing. The pasteurized liquid MCC, 3% (wt./wt.), was split into two equal parts. One part was utilized to produce C-MCC powder by ultrafiltering at a temperature of 20 °C, and a base, boost, and inlet pressure of 207, 138, and 345 kPa, respectively, using spiral wound membranes (Microdyn^®^ modules) of 20 kDa molecular weight cut-off and a total surface area of 5.7 m^2^. The remaining part of the pasteurized liquid MCC was utilized to produce RC-MCC powder following the UF process used during the control manufacturing process, except that the CO_2_ was injected into the liquid MCC before and during the UF processing step to reach and maintain a pH of 5.7 ± 0.1 before and during UF. The pH of CO_2_-treated UF retentates and the control UF retentates were adjusted to 7.0 ± 0.1 using 1.25 N NaOH and subsequently spray dried using a two-stage pilot scale spray dryer (Dahmes Stainless, New London, MN, USA) with a 21 core and 60 nozzle arrangement. The product was preheated to 35 ± 2 °C, and the feed pump pressure of 16.5 ± 0.7 mPa was maintained. The inlet and outlet air temperature were 190 ± 5 °C and 80 ± 5 °C, respectively. The powder manufacturing was performed in triplicates from three different lots of skim milk (approximately 748 kg).

### 2.2. Powder Characterization

#### 2.2.1. Chemical Composition

The C-MCC and RC-MCCs were analyzed using standard wet chemistry procedures for total solids (TS), total fat, and ash [53]. The lactose content was determined using an HPLC method [54]. Total nitrogen (TN), non-protein nitrogen protein (NPN), and non-casein nitrogen (NCN) were determined using micro-Kjeldahl analysis as described by Hooi et al., 2004, except the modified NCN extraction method developed by Zhang and Metzger, 2011 [55] was used. The true protein, casein, and whey protein were calculated by difference using the TN, NCN, and NPN values as described by Hooi et al., 2004. Mineral analysis of the samples was conducted using ICP-OES (inductive coupled plasma-optical emission spectroscopy). The proximate composition of each powder sample was determined at least in duplicates.

#### 2.2.2. Particle Size Distribution of MCC Powders

The particle size of the MCC powders was measured using a Malvern Mastersizer (Mastersizer3000; Malvern Instruments Ltd., Malvern, Worcestershire, UK) equipped with an Aero S dry dispersion unit. The refractive index of the sample and air was set at 1.45 and 1.00, respectively. The air pressure was set at 2 bar for all samples, and the feed rate was adjusted (from 25–100%) to accommodate the inherent variability of the cohesiveness of the powders at a 3 mm hopper gap. Size measurements were recorded as the median diameter (D50) and cumulative diameters (D90), where 50 and 90 refer to the percentile of the sample volume, with particle size less than the number indicated.

#### 2.2.3. Bulk Density

The bulk density of the MCC powder was measured for both loose and tapped conditions according to the IDF Standard 134A:1995. For the loose density, MCC powder was poured in a dry pre-weighed 100 mL calibrated glass cylinder up to the mark of 100 mL without any shaking and then weighed. After weighing, the same cylinder was tapped 100 times using UNILAB-009 Bulk Density Apparatus (Ambala Cantt, Haryana, India), and then volume after tapping was measured. Loose bulk density was calculated by dividing the weight of the powder by the volume of the powder before tapping. The tapped bulk density was calculated by dividing the weight of the powder by the volume of the powder after tapping. 

### 2.3. Functional Properties

The MCC dispersions (both control and Ca-reduced) were prepared as per the method outlined in the solubility test. In addition, dispersions after overnight storage were adjusted to a pH of 7.0 ± 0.1.

#### 2.3.1. Powder Solubility

The solubility of the MCC powders in room temperature deionized water was determined gravimetrically. The 5% protein solutions (wt./wt.) were made by dissolving measured MCC powders into the water at 22 ± 1 °C. The powders were stirred for 30 min into deionized water using a magnetic stirrer and a stir plate (Fisher Scientific) at a speed of 300 rpm. Post dissolution, the dispersions were hydrated overnight at 4 °C. Following overnight hydration, the protein solutions were allowed to equilibrate, and the pH was adjusted to 6.9 ± 0.1 using 2.0 and 0.2 N NaOH. The solubility method described by [56] was used with some modifications. Aliquots of each reconstituted MCC (50 mL) were centrifuged (CR4-12, Jouan Inc., Riverview, FL, USA) at 700× *g* for 10 min. The samples of the 5% (wt./wt.) protein solution (before centrifugation) and the supernatant collected after centrifugation were analyzed for TS. The TS was determined using a forced draft oven (Fisher Scientific) by drying at 100 °C for 4 h. The solubility of powder was calculated as the TS of supernatant, expressed as percentage of the TS of 5% protein solution prior to centrifugation. The changes in the solubility of the MCC powders during storage at 38 °C were further tested for four weeks. Each MCC sample was tested for solubility in duplicates.

#### 2.3.2. Apparent Viscosity

The apparent viscosity of the dispersions adjusted to pH 7.0 after overnight storage was determined at 20 °C using a rheometer (MCR 92, Anton Paar GmbH, Ostfildern, Germany). The dispersion was filled up to the mark into a concentric cylinder geometry consisting of a cup and bob. An equilibrium time of 25 s and a pre-shear rate of 10 for 20 s were applied. The solution viscosity was measured at a shear rate of 100/s. The viscosity data obtained are reported in centipoise (mPa-s). The viscosity measurements on each sample were tested in duplicates.

#### 2.3.3. Heat Stability

To determine heat stability of the MCC dispersions, an aliquot of 3 mL was transferred to a capped glass vial (61 mm height × 17 mm diameter.) and immersed in a clear mineral oil (99.9% mineral oil, USP, Vi-Jon, TN, USA) maintained at 140 °C in a bath (Akash-Deep Scientific Industries, New Delhi, India) with constant agitation. The heat coagulation time (HCT) was determined as the time in min elapsed between immersing the samples in the oil bath and the onset of visual clots [57].

#### 2.3.4. Emulsifying Capacity and Emulsion Stability

To evaluate the emulsion capacity and emulsion stability of MCC powders, an emulsion was prepared by mixing soyabean oil with freshly reconstituted MCC samples (1% wt./wt.) at the ratio of 3:7 (wt./wt.). Dispersion of MCC 80 (1%, wt./wt.) was prepared by adding MCC80 powder in deionized water and then stirred using a magnetic stirrer (700 rpm) for 60 min at 22 °C. The pH of the dispersions was adjusted in the range of 6.9 ± 0.1 using 2.0 and 0.2 N NaOH. Next, 7 g of reconstituted MCC dispersion was taken in a 50 mL centrifuge tube, and then 3 g soybean oil was added to it. The blend of MCC solution and oil was heated to 55 °C and homogenized for 60 s at 10,000 rpm using a benchtop homogenizer (Polytron, PT 2500E). Approximately 8 g of the emulsion was transferred to another 15 mL centrifuge tube, followed by centrifugation at 1100× *g* for 5 min. The height of the emulsified layer and the total contents in the tube were recorded. The emulsifying capacity was calculated as % of the volume of emulsified liquid to the total volume of the liquid homogenized using the below formula.

Emulsion Capacity, EA (%) = [H_E_/H_T_] ×100 (1)

where H_E_ is the height of the emulsified layer in the tube and H_T_ is the height of the total content in the tube.

To determine the emulsion stability, the emulsion prepared was heated to 80 °C for 30 min. in a water bath, then brought down to room temperature (22 °C) and recentrifuged at 1100× *g* for 5 min. The emulsion stability was calculated as % of the volume of emulsified liquid after heating to the total volume of the liquid homogenized using the below formula.

Emulsion stability, ES (%) = [H_H_/H_E_] × 100 (2)

where H_H_ is the height of the emulsified layer after heating, cooling, and re-centrifugation.

#### 2.3.5. Foaming Capacity (Overrun) and Foam Stability

Foaming capacity was determined using the method described by [58]. First, 3 g MCC80 powder was blended with 100 mL phosphate buffer (0.05 mol·L^−1^, pH 7.0) in a mixer (auto-mix Osterizer blender, Model: 6630) and whipped for 6 min at 11,000 rpm. The developed foam was immediately transferred into a 250 mL measuring cylinder quantitatively, and the total volume was recorded. The foaming capacity was calculated using the below equation.

Foam capacity, FC (%) = [(V_0_ − V_L_)/V_L_] × 100 (3)

where V_L_ is the volume of liquid before whipping (mL) and V_0_ is the total volume (foam plus liquid) obtained immediately after whipping (mL)

To determine the foam stability, the cylinder containing foam was kept undisturbed for 30 min at 22 °C. Then, the volume after the holding time was recorded. The foam stability was determined as the volume of foam that remained after 30 min (at 22 ± 1 °C) expressed as a percentage of the initial foam volume using the equation below.

Foam stability, FS (%) = [(V_T_ − V_L_)/(V_0_ − V_L_)] × 100 (4)

where V_T_ is the foam volume after 30 min of whipping.

### 2.4. Statistical Analysis

The treatments were run in triplicates, and the chemical analyses were run at least in duplicate. The values of the replicates are presented as mean ± standard deviation (SD). Using Minitab^®^ (v.20.4, Minitab Inc., State College, PA, USA), to assess for significant differences between the group means, a one-way analysis of variance (ANOVA) was used, followed by the Tukey post hoc comparison test. Statistical significance was defined as a *p*-value of less than 0.05.

## 3. Results and Discussion

### 3.1. Powder Characterization

The principal goal of this study was to produce an MCC powder with 30% calcium reduction (RC-MCC) by injecting CO_2_ and using membrane processing at a pilot scale (Figure 1). The general composition and the mineral concentration of C-MCC powders thus produced are summarized in Table 1 and Table 2. The total protein content of the C-MCC and RC-MCC powders was ~80% (wt./wt.), qualifying the product as MCC80. There was no significant difference between the true protein content (and the casein) in C-MCC and RC-MCC, and the results were 77.81% (71.74%) and 78.99% (72.16%), respectively. Similarly, there were no significant differences in the rest of the general chemical composition, i.e., total solids, whey protein, fat, and lactose. However, using a novel MF-UF process and injecting CO_2_ in the treatment, the calcium content was significantly reduced in the RC-MCC powder (31.01%) compared to control MCC powders. The ash, calcium, and phosphorus contents were significantly lower in RC-MCC, 7.06%, 1727 mg/100 g, and 1267 mg/100 g, respectively, compared to MCC, 8.15%, 2503 mg/100 g, and 1563 mg/100 g, respectively. The significant increase in sodium content in RC-MCC powders was due to the pH adjustment of liquid RC-MCC using 5% wt./wt. NaOH solution prior to drying. The equilibrium between colloidal and soluble Ca depends on the pH of the milk system [59,60]. During the acidification process of milk, the lowering of pH causes the serum phase to become less saturated in calcium phosphate due to the dissociation of this salt; consequently, the micellar calcium phosphate is progressively dissolved with an increase in the amounts of calcium and inorganic phosphate concentrations in the aqueous phase [61,62,63,64]. During the UF process employed in the manufacturing of RC-MCC, the soluble minerals portioned into the permeate, resulting in a significant reduction in calcium (31%) and phosphorus (19%) in the retentate.

The physical characteristics of the MCC powders, i.e., particle size and bulk density, are summarized in Table 3. The particle size distribution, [D,90] values of RC-MCC (65.77) were significantly lower than that of C-MCC (73.97). Colloidal calcium phosphate (CCP) plays an essential role in the stability and the structural integrity of casein micelles, and it exists in equilibrium with the Ca present in the serum phase [64]. Therefore, when the skim milk system is acidified, the CCP from the micelles moves into the serum phase. With progressive removal of CCP during acidification of skim milk, the casein micelles become smaller and more homogeneous [65]. A recent study reported that a 33% reduction of calcium in MCC by chelating calcium using Na_2_HPO_4_ resulted in the decrease in the casein micelle size [66]. A similar effect on the micellar size was observed when calcium was depleted by adding other chelating agents, and an increasing micellar size was reported when calcium was added [49,67,68]. The reduced particle size in RC-MCCs decreased both tapped and loose bulk density parameters. However, the difference was significant in the case of tapped bulk density (Table 3). This is because the smaller particles were much lighter and had much higher specific surface areas and thus trapped air much more efficiently in a bed of powder, which reduced the bulk density [69].

### 3.2. Functional Properties of MCC Dispersions

The results of different functional properties, i.e., solubility, viscosity, HCT, emulsification capacity, emulsion stability, foam capacity, and foam stability of MCC and RC-MCC are summarized in Table 4.

#### 3.2.1. Solubility

The solubility of MCC and RC-MCC were compared using the suspensions of fresh powders and the powders stored at elevated temperatures (38 °C) for four weeks to understand and compare the impact of calcium depletion on the solubility of MCC powders during storage. The solubility of the MCC and RC-MCC powders are presented in Table 4. The solubility of RC-MCC dispersions (99.36%) was observed to be significantly (*p* < 0.05) higher than C-MCC (88.82%). A similar effect of reducing the pH of skim milk during ultrafiltration to manufacture reduced calcium MPC powders on the solubility of resulting MPC dispersions was reported by Liu et al., 2019 [70] and Marella et al., 2015. Furthermore, Schäfer et al., 2021 reported a significant improvement in the solubility of reduced-calcium MCC powder dispersions. However, the method and expression of the solubility that we used in this experiment were different from the one used by Schäfer et al., 2021. They used the ADPI solubility method, and the solubility was expressed as the amount of insoluble material. In contrast, we used the gravimetric solubility method as described in the materials and methods section. Another reason for the difference in the solubility numbers between our test and those of Schäfer et al., 2021 is that they dried the calcium-reduced MCCs at an unadjusted and notably lower pH (5.8). In contrast, in our processing, the pH of the RC-MCC liquids was adjusted to ~7.0 ± 0.1 before drying to avoid any unwanted buildup of viscosity, and clogging of the lines and nozzles was observed during the initial trials of the manufacturing. This improved solubility of RC-MCC powders could be explained by the increased nonmicellar casein fraction in RC-MCC powders, which was not susceptible to the development of insolubility during drying [71]. Additionally, the increase in non-micellar casein fractions and the net zeta potential of the casein micelles (data not shown) might have prevented the aggregation of micellar casein in RC-MCC dispersions. In summary, the smaller particle size distribution, high surface negative electrostatic repulsions, and low ionic calcium activity [42,68] of the neutralized RC-MCC dispersions were the principal reasons for the improved solubility in the case of calcium-reduced micellar casein powders compared to those of control MCC powders.

**Figure 2:**
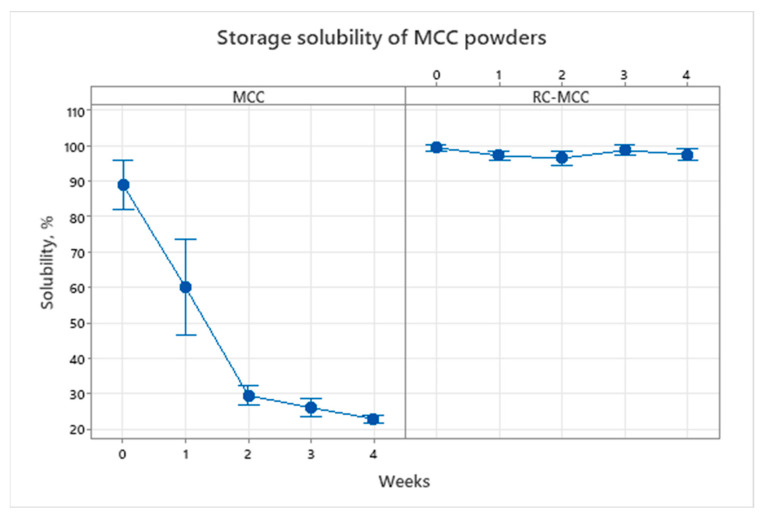
The solubility of MCC and RC-MCC dispersions, stored at 38 °C for 30 d (four weeks).

It was theorized that during the storage of MCC powders, the progression of protein-protein interactions and cross linkage resulting in increased aggregation causes a decrease in solubility [56,72]. Several other studies [20,21,23,24,43,44,47,73,74,75,76,77] have reported improved initial solubility of calcium-reduced high-casein-containing powders (MPCs and MCCs); however, there was no information available on the impact of calcium reduction on the storage solubility of RC-MCC powders. The change in the solubility of MCC and RC-MCC dispersions is presented in Figure 2. The RC-MCC powders were observed to retain their solubility for the duration of the storage period, and there was no significant drop in the solubility up to 30 d of storage at 38 °C. The control MCC dispersions showed a significant loss (approximately 30%) in solubility in the first week of storage itself. Toward the end of the storage period (30d), about 65% of the solubility was lost in the case of C-MCC, whereas an insignificant loss of 2% was observed with RC-MCC. As the storage time of MCC progresses, the hydrophobic protein–protein interactions result in a network of casein micelles via non-covalent bonding at the powder particle surface, causing the solubility deterioration [78]. In the case of control MCC, the initial solubility of 89% was similar to that of the non-calcium adjusted powders, dried after adjusting the retentate pH to 6.7 prior to drying, as reported by Liu et al., 2019. Likewise, the loss in solubility after three weeks of storage at 38 °C in the case of control MCC (26%, solubility) was comparable to 19% solubility after a storage period of 84 d at 40 °C. The loss in storage stability can be reduced when skim milk is acidified to deplete the calcium prior to membrane filtration and spray drying [46,70]. Meanwhile, the calcium depleted RC-MCC powder showed 99% initial solubility and 98% solubility toward the end of the storage period of 3 weeks at 38 °C, retaining almost all the initial solubility. These findings are consistent with those of a previous study, wherein the skim milk was acidified using citric acid (from a pH of 6.8 to 5.9) before spray drying [79].

#### 3.2.2. Viscosity

The viscosity of the reconstituted 5% (wt./wt.) solutions of MCC and RC-MCC powders at 20 °C, 2.37 mPa-s and 2.46 mPa-s, respectively (Table 4), were found to be non-significant (*p* > 0.05). Nevertheless, the RC-MCC dispersions had higher apparent viscosities, and this could be attributed to increased voluminosity of casein micelles due to a decrease in the colloidal calcium phosphate [80]. Schäfer et al., 2019 reported no significant difference in the viscosity between control MCC and 50% calcium-reduced MCC powder dispersion.

#### 3.2.3. Heat Stability

The casein micelles in milk are remarkably stable systems that can withstand the rigorous conditions applied during the commercial sterilization conditions [29]. However, the heat stability of micellar casein is significantly influenced by the mineral equilibrium [30]. The heat stability of RC-MCC powder dispersions was observed to be significantly higher (*p* < 0.05), 26.47 min., compared to 10.62 min for the control MCC dispersion (Table 4). This was a noticeable improvement, and approximately 2.5 times higher heat stability of MPCC dispersions could be achieved by reducing 30% calcium. A similar effect of calcium reduction on the MPC dispersions was reported by Sunkesula et al., 2021. They studied the effect of calcium reduction in MPCs on the heat stability of reduced-calcium MPC dispersions at different adjusted pH values. They reported that a 30% reduction of calcium resulted in a significant improvement in the heat coagulation time (HCT) of the dispersions at a pH of 6.9 ± 0.1. They summarized that the heat stability of reduced-calcium casein dispersions is an interplay of colloidal reactions affected by calcium ion activity, ionic composition, and dissociation of caseins, which affects the aggregation behavior of caseins during heating. Therefore, the observed improved heat stability of the RC-MCC dispersion could be attributed to the same phenomenon.

#### 3.2.4. Emulsification Capacity and Emulsion Stability

An emulsion is generally described as a mixture of two immiscible liquids (for example, water and oil), wherein one of these liquids is dispersed as droplets in the other [81]. Most food systems commonly contain particulate material that accumulates at oil–water and air–water interfaces and contributes to the colloidal stabilization of emulsions and foams [82]. The emulsifying agents are adsorbed at the oil–water interface and reduce surface tension, thus stabilizing the emulsion [83]. Caseins can be adsorbed at the interface, either in individual or aggregated form [84], hence functioning as an emulsifying agent. Among the functional properties, the casein micelle’s ability to emulsify and stabilize oil in water emulsions is of great interest to the food industry, particularly the dairy industry.

The C-MCC and RC-MCC powders were assessed for their ability to facilitate the blending of the phases of the emulsion (emulsification capacity) and their ability to stabilize the emulsion (emulsion stability). The results are presented in Table 4. The emulsification capacity of the RC-MCC (66%) powders was observed to be higher than that of the control MCC (63%). The differences in the emulsification capacity could generally be attributed to the surface activity and/or the size of the emulsifying agent; the higher the surface activity and/or the smaller the size, the greater the emulsification capacity. The higher zeta potential (data not shown) and lower particle size distribution of RC-MCC compared to those of the control MCC powders could have affected the emulsification capacity of RC-MCC powders positively. Similar results were reported by Lazzaro et al., 2017 where the emulsifying capacity and stability of casein aggregates were characterized after sequential calcium and simultaneous inorganic phosphorus depletion using trisodium citrate (TSC). However, the emulsification stability of the RC-MCC (81%) powders was observed to be significantly (*p* < 0.05) lower than the control MCC (87%). Lazzaro et al., 2017 proposed that the destabilization of emulsions can result from three phenomena, i.e., creaming, flocculation, and coalescence. They observed that the emulsions stabilized by calcium-depleted casein were stable against coalescence but not stable against creaming and flocculation phenomena. The creaming and flocculation are two concomitant phenomena and influence each other. Creaming happens in the emulsions due to the difference in the density between the oil and the aqueous suspension phases and is enhanced as the oil droplet aggregation progresses. The creaming, on the other hand, facilitates flocculation by moving the droplets forward and encouraging contact, which is a critical step in the ultimate instability of emulsion [85]. The presence of unadsorbed particles causes depletion flocculation, an instability process that happens in emulsions. It occurs when two neighboring droplets are close enough to exclude any unadsorbed particles from the gap separating them. As a result, an osmotic pressure differential is created, which causes the emulsion droplets to attract each other [86,87]. Ye et al., 2013 reported the effect of depletion flocculation on decreasing the emulsion stability in calcium-depleted MPC stabilized emulsions. This could explain the lower emulsification stability of the RC-MCC powders compared to that of control C-MCC powders. 

#### 3.2.5. Foaming Capacity and Foam Stability

The foaming properties of proteins are related to the adsorption (surface activity) of proteins to the air/aqueous surface, i.e., the rate at which the surface tension of the air–water interface decreases. The foaming performances are influenced by factors such as the type and concentration of protein, temperature, pH, ionic environment, ionic strength, and conformation of these proteins [82,88,89]. The addition of calcium chelating agents such as ethylenediaminetetraacetic acid (EDTA) causes dissociation of casein micelles, and the higher availability of β-casein would be preferentially adsorbed onto the foam, thus improving the foamability of protein dispersions [90]. Another study demonstrated different degrees of calcium demineralization by acidification using HCl and ultrafiltration of milk and reported that 9.5% wt./wt. milk protein dispersions thus obtained showed an increased β-casein and non sedimental casein fraction with the increase in acidification [91]. As shown in Table 4, the 3% wt./wt. RC-MCC dispersions showed a significantly high foaming capacity (*p* < 0.05) compared to the C-MCC dispersions. When CO_2_ was injected into liquid MCC before and during ultrafiltration to adjust the pH to ~5.7, the dissociation of casein micelles and increased nonmicellar casein (β-casein, in particular) could have improved the foaming capacity of RC-MCC dispersions. These results align with the finding of Silva et al., 2013. However, the foam stability (Table 4) of RC-MCC dispersions was significantly lower than that of C-MCC dispersions. One of the important physical properties of the dispersions that helps retain the stable foam is viscosity. Higher viscosities promote the formation of smaller air bubbles and reduce the coalescence of air bubbles, thereby enhancing the stability of the resulting foam [92]. Given that there was no significant difference between the viscosities of C-MCC and RC-MCC dispersions and more foam incorporated in the RC-MCC dispersions, the RC-MCC dispersions showed less foam stability than C-MCC dispersions.

## 4. Conclusions

This study evaluated the pilot-scale production of calcium-reduced MCC 80 powders using a novel microfiltration–CO_2_ injection–ultrafiltration process and the effect of the calcium reduction on the physicochemical and functional properties of the RC-MCC powders and dispersions. In addition, control micellar casein powders (C-MCC) without CO_2_ injection were also produced to compare with RC-MCC. From our investigation, we concluded the following:

i. A 30% calcium reduction in MCC powders may be feasible at a commercial scale using CO_2_ injection. Further, using CO_2_ is a cleaner process than using acidulants to lower the pH of milk or microfiltered milk to reduce the calcium content because residual CO_2_ could be easily removed either by applying vacuum or heat.
ii. Reducing the calcium content of MCC decreased the particle size and bulk density of the resultant powders. This could be attributed to the dissociation of casein micelles during CO_2_ injection. This significantly improved the instant solubility, and the lower calcium levels retained the solubility of the RC-MCC powders. The reduction in calcium also improved the heat stability of the dispersions.
iii. Reduction in calcium was observed to improve foam capacity; however, the emulsions stability and foam stability were lower than control powder dispersions. This could be attributed to smaller particle size and not enough viscosity to retard the coalescence of smaller oil droplets or foam bubbles.

These research findings add to the current understanding of the functionality of reduced-calcium micellar casein powders and help users apply MCC powder ingredients more effectively. 

## Figures and Tables

**Table 1:** Mean (n = 3) chemical composition of the micellar casein concentrate powders (% wt./wt.).

Powder ^1^	Composition							
	Total Solids	Moisture	Total Protein	True Protein	Casein	Whey Protein	Fat	Lactose
C-MCC	95.51 ± 0.11 ^a^	4.49 ± 0.11 ^a^	79.25 ± 0.82 ^a^	77.81 ± 0.88 ^a^	71.74 ± 1.13 ^a^	6.07 ± 0.54 ^a^	2.98 ± 0.41 ^a^	5.13 ± 0.94 ^a^
RC-MCC	95.34 ± 0.27 ^a^	4.62 ± 0.27 ^a^	80.33 ± 0.72 ^a^	80.00 ± 0.51 ^a^	72.16 ± 0.40 ^a^	6.83 ± 0.30 ^a^	2.91 ± 0.61 ^a^	5.08 ± 0.62 ^a^

^1^ Abbreviations are: C-MCC, control micellar casein concentrate powder; RC-MCC, calcium-reduced micellar casein concentrate powder. ^a^ Mean ± SD values are not significantly different (*p* < 0.05).

**Table 2:** Mean (n = 3) mineral composition of the micellar casein concentrate.

Powder ^1^	Composition				
	Ash, %	Na, mg/100 g	P, mg/100 g	Ca, mg/100 g	Ca Reduction, %
C-MCC	8.15 ± 0.21 ^a^	169 ± 13 ^a^	1563 ± 60 ^a^	2503 ± 90 ^a^	−
RC-MCC	7.06 ± 0.42 ^b^	700 ± 80 ^b^	1267 ± 50 ^b^	1727 ± 100 ^b^	31.01 ± 3.39

^1^ Abbreviations are: C-MCC, control micellar casein concentrate powder; RC-MCC, calcium-reduced micellar casein concentrate powder, Na, sodium; P, phosphorus; Ca, calcium. ^a,b^ Mean ± SD values not sharing a common superscript within the same column are significantly different (*p* < 0.05).

**Table 3:** Physical characteristics of the micellar casein concentrate (n = 3).

Powder ^1^	Physical Property			
	Particle Size, [D,50]	Particle Size, [D,90]	BD-Untapped, kg/m^3^	BD-Tapped, kg/m^3^
C-MCC	33.02 ± 2.88 ^a^	73.97 ± 3.24 ^a^	181.34 ± 12.17 ^a^	304.14 ± 13.81 ^a^
RC-MCC	33.72 ± 1.22 ^a^	65.77 ± 2.68 ^b^	160.27 ± 5.45 ^a^	271.31 ± 7.93 ^b^

^1^ Abbreviations are: C-MCC, control micellar casein concentrate powder; RC-MCC, BD, bulk density. ^a,b^ Mean ± SD values not sharing a common superscript within the same column are significantly different (*p* < 0.05).

**Table 4:** Functional characteristics of the micellar casein concentrate (n = 3).

Powder ^1^	Functional property						
	Powder Solubility, %	Viscosity, mPa-s	HCT, min.	Emulsification Capacity, %	Emulsion Stability, %	Foam Capacity, %	Foam Stability, %
C-MCC	88.82 ± 2.77 ^a^	2.37 ± 0.10 ^a^	10.62 ± 0.47 ^a^	63.33 ± 0.87 ^a^	86.48 ± 1.79 ^a^	102.67 ± 6.11 ^a^	94.54 ± 2.08 ^a^
RC-MCC	99.36 ± 0.35 ^b^	2.46 ± 0.10 ^a^	26.47 ±0.60 ^b^	65.97 ± 0.94 ^a^	80.97 ± 1.07 ^b^	126.50 ± 3.62 ^b^	92.51± 1.65 ^b^

^1^ Abbreviations are: C-MCC, control micellar casein concentrate powder; RC-MCC, HCT, heat coagulation time. ^a,b^ Mean ± SD values not sharing a common superscript within the same column are significantly different (*p* < 0.05).

## Data Availability

Data is contained within the article.
